# Adaptive Mistranslation Accelerates the Evolution of Fluconazole Resistance and Induces Major Genomic and Gene Expression Alterations in *Candida albicans*

**DOI:** 10.1128/mSphere.00167-17

**Published:** 2017-08-09

**Authors:** Tobias Weil, Rodrigo Santamaría, Wanseon Lee, Johan Rung, Noemi Tocci, Darren Abbey, Ana R. Bezerra, Laura Carreto, Gabriela R. Moura, Mónica Bayés, Ivo G. Gut, Attila Csikasz-Nagy, Duccio Cavalieri, Judith Berman, Manuel A. S. Santos

**Affiliations:** a Department of Medical Sciences & Institute of Biomedicine, iBiMED, University of Aveiro, Aveiro, Portugal; b Research and Innovation Centre, Fondazione E. Mach, San Michele All’Adige, Italy; c Department of Computer Science, University of Salamanca, Salamanca, Spain; d European Molecular Biology Laboratory, European Bioinformatics Institute (EMBL-EBI), Wellcome Trust Genome Campus, Hinxton, United Kingdom; e Wellcome Trust Centre for Human Genetics, University of Oxford, Roosvelt Drive, Oxford, United Kingdom; f SciLifeLab, Uppsala University, Uppsala, Sweden; g Department of Genetics, Cell Biology and Development, University of Minnesota, Minneapolis, Minnesota, USA; h Centro Nacional de Análisis Genómico, Parc Científic, Barcelona, Spain; i Randall Division of Cell and Molecular Biophysics and Institute for Mathematical and Molecular Biomedicine, King’s College London, London, United Kingdom; j Department of Molecular Microbiology and Biotechnology, Tel Aviv University, Ramat Aviv, Israel; Carnegie Mellon University

**Keywords:** *Candida albicans*, fluconazole, LOH, aneuploidy, codon ambiguity, drug resistance evolution, phenotypic variability, protein mistranslation

## Abstract

Regulated erroneous protein translation (adaptive mistranslation) increases proteome diversity and produces advantageous phenotypic variability in the human pathogen *Candida albicans*. It also increases fitness in the presence of fluconazole, but the underlying molecular mechanism is not understood. To address this question, we evolved hypermistranslating and wild-type strains in the absence and presence of fluconazole and compared their fluconazole tolerance and resistance trajectories during evolution. The data show that mistranslation increases tolerance and accelerates the acquisition of resistance to fluconazole. Genome sequencing, array-based comparative genome analysis, and gene expression profiling revealed that during the course of evolution in fluconazole, the range of mutational and gene deregulation differences was distinctively different and broader in the hypermistranslating strain, including multiple chromosome duplications, partial chromosome deletions, and polyploidy. Especially, the increased accumulation of loss-of-heterozygosity events, aneuploidy, translational and cell surface modifications, and differences in drug efflux seem to mediate more rapid drug resistance acquisition under mistranslation. Our observations support a pivotal role for adaptive mistranslation in the evolution of drug resistance in *C. albicans*.

**IMPORTANCE** Infectious diseases caused by drug-resistant fungi are an increasing threat to public health because of the high mortality rates and high costs associated with treatment. Thus, understanding of the molecular mechanisms of drug resistance is of crucial interest for the medical community. Here we investigated the role of regulated protein mistranslation, a characteristic mechanism used by *C. albicans* to diversify its proteome, in the evolution of fluconazole resistance. Such codon ambiguity is usually considered highly deleterious, yet recent studies found that mistranslation can boost adaptation in stressful environments. Our data reveal that CUG ambiguity diversifies the genome in multiple ways and that the full spectrum of drug resistance mechanisms in *C. albicans* goes beyond the traditional pathways that either regulate drug efflux or alter the interactions of drugs with their targets. The present work opens new avenues to understand the molecular and genetic basis of microbial drug resistance.

## Abstract (precis)

Infectious diseases caused by drug-resistant fungi are an increasing threat to public health because of the high mortality rates and high costs associated with treatment. Thus, understanding of the molecular mechanisms of drug resistance is of crucial interest for the medical community. Here we investigated the role of regulated protein mistranslation, a characteristic mechanism used by *C. albicans* to diversify its proteome, in the evolution of fluconazole resistance. Such codon ambiguity is usually considered highly deleterious, yet recent studies found that mistranslation can boost adaptation in stressful environments. Our data reveal that CUG ambiguity diversifies the genome in multiple ways and that the full spectrum of drug resistance mechanisms in *C. albicans* goes beyond the traditional pathways that either regulate drug efflux or alter the interactions of drugs with their targets. The present work opens new avenues by which to understand the molecular and genetic basis of microbial drug resistance.

## INTRODUCTION

Fungal infections are an increasingly serious health problem because of immune deficiencies caused by diseases like HIV/AIDS and cancer therapies, as well as prolonged antibiotic treatments (1). Deep-seated and disseminated infections are difficult and extremely costly to treat and are normally associated with high mortality rates. In the United States alone, the costs of antifungal therapy have reached $8 billion/annum (2). Resistance to the commonly used azoles is increasing, and alternative antifungals, such as encapsulated amphotericin B or echinocandins, are expensive and increase the cost of antifungal therapy dramatically.

Antifungal resistance has been studied intensively and involves DNA mutations, genome plasticity, cell signaling, and gene expression alterations (3–5). In the specific case of azoles, resistance is due mainly to mutations in the ergosterol biosynthesis pathway targeted by the drug and drug efflux. Lanosterol 14α-demethylase (encoded by *ERG11*) is a key enzyme in the ergosterol biosynthesis pathway and the direct target of azole antifungals. Overexpression of *UPC2*, the transcription factor regulating *ERG11*, or aneuploidy events that increase the *ERG11* copy number lead to increased azole resistance (6, 7). Gain-of-function mutations in *TAC1* and *MRR1* (7, 8), transcription factors that positively regulate the transcription of efflux pumps encoded by the *CDR1*/*CDR2* or *MDR1* genes (5, 9), also result in increased drug resistance. Types of mutations that result in acquired resistance include genome rearrangements, protein sequence changes (4, 5), aneuploidy and loss of heterozygosity (LOH) (6, 10–13), and increased mutagenesis due to loss of mismatch repair or elevated levels of DNA double-strand break repair (3).

*Candida albicans*, the most prevalent pathogen of humans, has a unique codon usage system whereby translational fidelity is modulated by environmental cues (14). A mutant serine tRNA is recognized and aminoacylated by both leucyl- and seryl-tRNA synthetases (LeuRS and SerRS, respectively) (15), yielding a charged tRNA that can incorporate either Leu or Ser at CUG codons. This leads to proteome-wide plasticity, such that each protein in the cell is represented by a mixture of polypeptides containing Ser or Leu at such sites. The basal level of Ser and Leu incorporated at CUG sites (3% leucine and 97% serine in rich medium) differs in different ecological niches and under different environmental conditions, contributing to proteomic and phenotypic diversification (16). In a previous study, we increased Leu misincorporation at CUG sites to 22% by inserting a copy of a yeast Leu tDNA_CAG_^Leu^ gene into the genome of *C. albicans* strain SN148 and could show that this strain was slightly more tolerant to fluconazole than the wild-type (WT) strain when grown on solid agar plates containing this antifungal (16). This result suggested that CUG mistranslation might have a role in antifungal drug tolerance. However, the underlying molecular mechanism by which proteomic plasticity results in fluconazole resistance is unknown. As mistranslation is associated with antibiotic resistance in bacteria (17–19), we hypothesized that it may also be relevant to the evolution of fluconazole resistance in *C. albicans*.

In the present study, we investigated how CUG mistranslation contributes to the acquisition of fluconazole resistance by evolving WT and hypermistranslating strains of *C. albicans* in parallel in the absence and presence of fluconazole. These strains were then characterized by genome resequencing, array-based comparative genome hybridization (CGH), and gene expression analysis. We found that mistranslation accelerated the acquisition of fluconazole resistance in *C. albicans* and revealed a number of genomic features and key genes that provide insights into how CUG mistranslation promotes resistance to fluconazole.

## RESULTS

**FIG 1:**
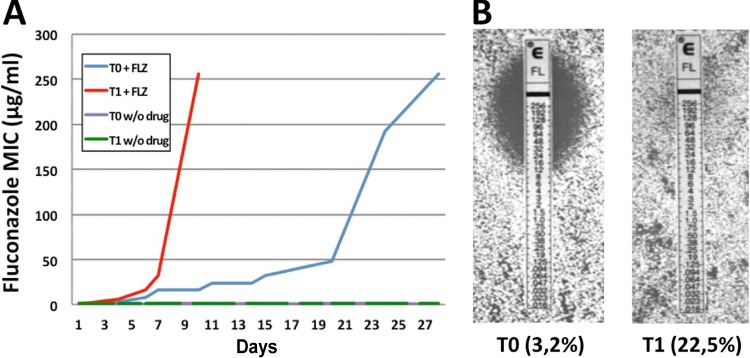
Fluconazole resistance profiles during evolution. (A) During evolution with fluconazole (FLZ), control strain TO (3.2% natural mistranslation; blue line) and hypermistranslating strain T1 (22.5% leucine misincorporation; red line) showed increased resistance to the drug over time yet revealed marked differences in the speed of resistance acquisition. Both strains evolved without (w/o) the drug (green and gray lines) did not show increased resistance. (B) E tests after 10 days of evolution. Hypermistranslating strain T1 rapidly adapted to the drug and showed complete resistance to fluconazole, while WT strain T0 was still sensitive.

To clarify the role of mistranslation in fluconazole resistance, we used *C. albicans* strains that incorporate Leu at CUG sites at different levels; a control strain (T0) incorporates the WT levels of 3% Leu and 97% Ser, and a hypermistranslating strain (T1) incorporates 22 and 78% Leu and Ser at the same sites, respectively (16). We asked initially if mistranslation alters the frequency of acquisition of fluconazole resistance during evolution. For this, strains T0 and T1 were evolved by serial passage in increasing concentrations of fluconazole (Fig. 1; see Fig. S1 in the supplemental material) and genetically tested at three different time points, i.e., at the beginning of the experiment (no fluconazole, no suffix), at medium fluconazole concentrations (16 to 32 µg/ml, suffix FM), and at a high fluconazole concentration (256 µg/ml, suffix FH) (Fig. 1; Fig. S1). Both strains were also evolved in the absence of fluconazole to test for effects due to mistranslation alone (suffix NF).

10.1128/mSphere.00167-17.1 FIG S1  Experimental design used in this study. RNA for gene expression analysis was extracted from growth duplicates of the same strains (Tx, TxFM, and TxFH) of which the whole genome was sequenced (WGS; genomic DNA extraction). Additionally, genomic DNA of three T1FH clones was extracted for array-based CGH analysis. The intensity of the red color of arrows indicates increasing fluconazole concentrations. Download FIG S1, EPS file, 0.4 MB. Copyright © 2017 Weil et al. 2017 Weil et al. This content is distributed under the terms of the Creative Commons Attribution 4.0 International license.

When evolved without fluconazole, the T0NF and T1NF strains did not exhibit a visible difference in resistance (Fig. 1, green and gray lines). In contrast, the evolution of either strain T0 or T1 in the presence of fluconazole resulted in the acquisition of high-level resistance (MIC of 256 μg/ml) during evolution with no difference in the mutation rate, albeit with a marked difference in the speed of acquisition. The hypermistranslating T1 strain became resistant (T1FH, MIC of 256 μg/ml) within 10 days of passaging (~2 days per passage), while control strain T0 began to show increased resistance after 1 week of evolution (T0FM) and reached a MIC of 256 µg/ml (T0FH) after 1 month of passaging (Fig. 1). Thus, increased levels of mistranslation appeared to speed up the emergence of fluconazole resistance. To address the generality of this phenomenon, we asked if mistranslation also promotes resistance to caspofungin, an antifungal drug of clinical relevance belonging to the echinocandin class. While the MICs for both strains T0 and T1 were in the same range (0.094 to 0.125 µg/ml), only the T1 hypermistranslating strain produced microcolonies within the entire inhibition ellipse of the E test. These microcolonies also grew in liquid medium containing caspofungin concentrations of up to 32 µg/ml (Fig. S2).

10.1128/mSphere.00167-17.2 FIG S2  Caspofungin susceptibility test. E tests of strains T0 and T1 revealed similar MICs (0.094 to 0.125 µg/ml), yet only hypermistranslating strain T1 produced microcolonies within the entire inhibition ellipse. These microcolonies also grew in liquid medium containing caspofungin concentrations of up to 32 µg/ml. Download FIG S2, EPS file, 0.8 MB. Copyright © 2017 Weil et al. 2017 Weil et al. This content is distributed under the terms of the Creative Commons Attribution 4.0 International license.

### Differences in resistance to fluconazole.

To clarify how resistance to fluconazole evolved in both strains T0 and T1, we resequenced the genomes of the evolved strains by using the Illumina sequencing platform and analyzed gene expression with the Agilent DNA microarray platform. The T0 strain acquired a known gain-of-function mutation (V877F) in transcription factor *MRR1* (20), a positive transcriptional regulator of the multidrug efflux pump gene *MDR1*; consistent with this, *MDR1* expression was upregulated 5.3-fold (Table S1; Fig. S3). The hypermistranslating T1 strain was remarkably different; it acquired a previously described A736V gain-of-function mutation in the transcriptional activator *TAC1*, which drives high levels of expression of the *CDR1* and *CDR2* genes, which encode the ABC transporters (21) (*CDR1*, 4.8-fold; *CDR2*, 3.9-fold; Table S1; Fig. S3). Analysis of the genomic DNA revealed LOH of *ERG11*, the target of fluconazole, in T1FM and T1FH; no such LOH event was observed in T0FM and T0FH. In addition, a previously unknown mutation (S35C) in the oxidosqualene cyclase gene *ERG7* was also detected in T0FM, T0FH, and T1FM (Table S1). Moreover, the expression of genes encoding components of the ergosterol biosynthesis pathway, the molecular target of fluconazole, were upregulated in both T0 and T1 fluconazole-resistant strains (Fig. S3). Taken together, these results suggest that both strains activated the common mechanisms of azole resistance, yet they did so via different drug efflux pathways and through different types of genomic alterations.

10.1128/mSphere.00167-17.3 FIG S3  The left panel is a schema depicting major events that allow strain T1 to acquire fluconazole resistance rapidly. Drug efflux in strain T1 seems to be mediated mainly via the ABC superfamily transporters *CDR1* and *CDR2*, while in strain T0, drug efflux is likely due to the multidrug pump encoded by *MDR1*. The right panel shows GO BP heatmaps that depict log-fold changes in gene expression. Green letters represent DEGs in T1FH/T1NF, i.e., those involved in steroid biosynthesis, glucose transport, and the chitin biosynthetic process. Download FIG S3, EPS file, 1.4 MB. Copyright © 2017 Weil et al. 2017 Weil et al. This content is distributed under the terms of the Creative Commons Attribution 4.0 International license.

10.1128/mSphere.00167-17.8 TABLE S1  Nonsynonymous codon changes in *TAC1*, *MRR1*, and *ERG7*. N.ofAA, position of amino acid; T0.Codon, codon in control strain T0; T0.AA, amino acid in control strain T0; S.Codon, codon in the strain tested; S.AA, amino acid in the strain tested. Download TABLE S1, DOCX file, 0.02 MB. Copyright © 2017 Weil et al. 2017 Weil et al. This content is distributed under the terms of the Creative Commons Attribution 4.0 International license.

### Genomic alterations induced by mistranslation and fluconazole.

A combination of whole-genome sequencing (WGS) and high-density array-based CGH data analysis provided deeper insight into the role of mistranslation in genome evolution.

By comparing genotype changes that were heterozygous in the nonevolved control strain and homozygous in the other samples analyzed, we found that strain T0 accumulated a moderate number of such events (<500) during evolution, regardless of whether fluconazole was present (T0FM and T0FH) or absent (T0NF). In contrast, the hypermistranslator T1 accumulated 2,193 LOH events during evolution in the absence of fluconazole (T1NF) and >5,100 LOH events in the presence of fluconazole (T1FM and T1FH). Thus, mistranslation alone resulted in the accumulation of a higher number of LOH events during evolution and the combination of mistranslation and fluconazole (strain T1) more than doubled the number of LOH events. Interestingly, in the resistant T1FH strain, a small group of open reading frames were especially rich in such LOH events, with orf19.2850 (yeast ortholog involved in telomeric silencing), *MEC1* (encodes a cell cycle checkpoint protein with a role in genome integrity), *HAL9* (yeast ortholog involved in salt tolerance), and *INT1* (has a role in morphogenesis and adhesion) having more than 51 LOH events (median, 5 LOH events).

Further, the evolution of the hypermistranslating T1 strain resulted in the appearance of two large LOH regions that include most of the above-mentioned events. A large LOH region on chromosome 5 (Chr5) appeared exclusively in the presence of fluconazole (T1FM and T1FH), while an LOH tract in the left arm of ChrR appeared regardless of drug treatment (T1NF, T1FM, and T1FH) (Fig. S4), suggesting that it was already present in the parental T1 strain or that it occurs at a recombination hotspot.

10.1128/mSphere.00167-17.4 FIG S4  LOH events in quickly evolving strain T1 (WGS data). While LOH on Chr5 appeared only under drug pressure (T1FM and T1FH), the LOH on the left arm of ChrR appeared regardless of fluconazole treatment (T1NF, T1FM, and T1FH). Download FIG S4, EPS file, 0.4 MB. Copyright © 2017 Weil et al. 2017 Weil et al. This content is distributed under the terms of the Creative Commons Attribution 4.0 International license.

**FIG 2:**
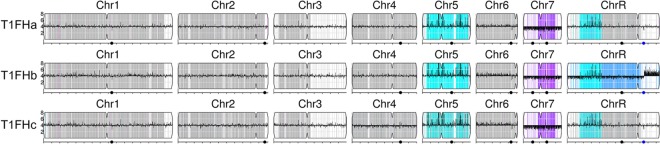
Y_MAP_ visualization of SNP-CGH data for three T1FH clones. The copy numbers and SNP-allele ratios of all three T1FH samples were visualized with Y_MAP_ (49). Changes in the copy number estimate for regions relative to the parental strain are illustrated by dark bars drawn up- or downward, depending on the direction and magnitude of the change. These strains appear to be tetraploid on the basis of the copy number estimates for Chr7 (and most of ChrR in clone T1FHb), which are 3/4 of the other chromosomes. Color illustrates SNP status across regions. Heterozygous regions are gray, white regions do not have SNPs in the SC5314 reference sequence, and cyan is homozygous “a” alleles (e.g., aaaa on Chr5 in all three clones and ChrR in T1FHa and T1FHc and aaa in T1FHb). Intermediate ratios are indicated by intermediate colors. Blue shade represents more copies of “a” alleles (e.g., aab on the central trisomic region of ChrR in T1FHb), and purple shade represents more copies of the “b” alleles (e.g., abb on Chr7 in all three clones). The portion of ChrR in clone T1FHb to the right of the rDNA region (blue dot) is present in five copies. Major repeat sequences are represented by black dots.

Notably, in both LOH regions, several loci encoding aminoacyl-tRNA synthetases and tRNA modification enzymes lost heterozygosity in strain T1 during evolution. We observed homozygosis of the cytosolic leucyl-tRNA synthetase gene locus (LeuRS; *CDC60*, ChrR) regardless of drug treatment. LeuRS charges the atypical tRNA_CAG_^Ser^ with leucine and is central to Leu/Ser ambiguity in mistranslation in *C. albicans* (22). Similar LOH events during evolution of strain T1 involved loci encoding the mitochondrial (*MSM1*) and cytosolic (*MES1*) methionyl-tRNA synthetases; the mitochondrial tryptophanyl-tRNA synthetase (*MSW1*); and the cytosolic phenylalanyl (*FRS1*)-, putative prolyl (*PRS*)-, putative aspartyl (*DPS1-1*)-, and tryptophanyl (*WRS1*)-tRNA synthetases; as well as orf19.3956 (orthologs have glutaminyl-tRNA synthase activity), orf19.2387 (a putative tRNA-Pro synthetase), and orf19.2382 (protein similar to isoleucyl-tRNA synthetase). Apart from *WRS1* (Chr1), these genes are all located within the LOH regions of ChrR and Chr5, suggesting that the tRNA aminoacylation system might be a driving force for the LOH on those chromosomes (Fig. 2; Fig. S4).

Since LOH events on Chr5 appear frequently in fluconazole-resistant *C. albicans* isolates (10), these data support the idea that mistranslation, LOH of Chr5, and fluconazole resistance are linked. To determine if genes affected by LOH events during evolution were selected because of their serine codon usage, we computed the relative synonymous codon usage (RSCU) of the seven serine codons of strains T1 and T1FH in all genes (with Anaconda [23]) and compared the two sets codon by codon. We found statistically significant differences in UCA, UCC, and UCG usage, which was decreased in the LOH-affected genes of the nonevolved T1 strain, and in UCG usage, which was decreased in the LOH-affected genes of T1FH (Table S2A).

10.1128/mSphere.00167-17.9 TABLE S2  CUG codon usage. (A) Statistical comparison of the altered set of genes for each evolutionary event and strain (T1 and T1FH genes affected by LOH and T1FH genes affected by copy number variations) and the rest of the genome. Significant results show that T1 genes affected by LOH are depleted of UCA, UCC, and UCG codons; T1FH genes affected by LOH are depleted of UCG codons; and T1FH genes affected by copy number variations are enriched in UCA codons and depleted of UCC and UCG codons. RSCU values for serine codons of each gene were calculated with Anaconda (23) and compared through Student *t* tests corrected by the Bonferroni method. (B) Numbers of CUG codons that differ in both alleles (a and b) in genes that lost heterozygosity or changed copy number in T1FH (data from CGD). Notably, the large LOH regions on Chr5 and ChrR are aaaa (see Fig. 2), which caused the highlighted changes in CTG codon usage. All of the genes that have a positive difference led to a reduction in CTG codons (blue) by changing CTG to other codons, while those having a negative difference increased the number of CTG codons (green) by changing other codons to CTG. Further, all of the genes with an increased copy number (red) had a positive difference, while those with a reduced copy number (orange) had a negative difference. Download TABLE S2, PDF file, 0.1 MB. Copyright © 2017 Weil et al. 2017 Weil et al. This content is distributed under the terms of the Creative Commons Attribution 4.0 International license.

Interestingly, the number of CUG codons differs between alleles (a and b) of genes that lost heterozygosity in T1FH (Table S2B); genes with a positive difference (a > b) underwent a reduction in the number of CTG codons, and genes with a negative difference (a < b) had an increase in the number of CTG codons. This suggests that the loss of one allele (Table S2B) had a balancing effect on the number of CTG codons.

As mentioned above, the LOH in the left arm of ChrR was present in all evolved T1 clones, regardless of whether cells were untreated (T1NF) or treated (T1FM and T1FH) with fluconazole (Fig. S4). The results imply that homozygosis in this region was already present as a standing variation in the starting population and was likely selected because of the presence of genes that are important in overcoming the potentially lethal effect of increased mistranslation. Because of their immediate availability and higher initial frequency, this standing variation (beneficial alleles) may speed up adaptation to new environments if compared to the time required for new beneficial mutations to arise (24). This LOH region on ChrR is enriched in genes with gene ontology (GO) terms related to peptide transport, cellular and nucleic acid metabolic processes, chromatin silencing at telomeres, and tRNA aminoacylation (Table S3). Of note, genes involved in transcriptional silencing by heterochromatin and aminoacylation were enriched among genes with sequence changes, and they represent two critical steps required for the accurate transfer of genetic information, a feature of particular importance under increased mistranslation.

10.1128/mSphere.00167-17.10 TABLE S3  GO BP categories of genes located within the region of ChrR that lost heterozygosity in all evolved T1 strains regardless of whether cells were untreated (T1NF) or treated with the drug (T1FM and T1FH). For genes with >10 LOH events, the corrected *P* value is <0.005. Download TABLE S3, DOCX file, 0.02 MB. Copyright © 2017 Weil et al. 2017 Weil et al. This content is distributed under the terms of the Creative Commons Attribution 4.0 International license.

### Copy number variation upon fluconazole exposure.

WGS revealed that the evolution of strain T1 in the presence of fluconazole also led to loss of chromosomal repeat regions in Chr6, Chr4, and Chr2 and of a few telomere-proximal genes (e.g., *CTA24*, *RRN3*, *TLO5*). Furthermore, it showed that evolution of strain T1 in the presence of fluconazole was accompanied by the apparent gain and loss of regions of Chr1, Chr4, Chr5, and ChrR. Specifically, T1FM had increased copies of Chr1 (164001 to 168200), Chr4 (46800 to 47200 and 518001 to 530000), Chr5 (744001 to 752000 and 860001 to 876000), and ChrR (1884001 to 1898000). Within these amplified chromosomal regions were, apart from the genes of the rDNA locus and repeat regions of Chr1, genes functioning in the regulation of transcription. Many of the amplified genome regions in strain T1FM were not detectable at the end of evolution in the presence of fluconazole. Rather, T1FH showed gains almost exclusively on Chr7 (150001 to 170000, 262001 to 328000, 318001 to 324000, 360001 to 380000, and 482001 to 576000), mainly located in the region between *WHI3* and *MRS7b*. The RSCU for serine codons in genes affected by copy number variation in T1FH showed increased usage of UCA and lower usage of UCC and UCG than the rest of the genome. Notably, the balancing effect of CUG codon usage observed for LOH was also detectable for copy number variation (Table S2).

To gain deeper insight into the segmental aneuploidies, we performed single nucleotide polymorphism (SNP)-CGH analysis of three T1FH clones (Fig. 2). Our CGH analysis suggested that all of our clones were nearly tetraploid, with Chr7 being trisomic. It appears that either the b homolog reduplicated or one copy of the a homolog was lost, leading to the Chr7 abb trisomy in T1FH (Fig. 2). One of the T1FH clones (T1FHb) was also trisomic (aaa) for the common LOH region of ChrR for which the other two clones were tetraploid (aaaa). In the T1FHb clone, the LOH region (aaa) of ChrR was followed by an additional trisomic LOH region (abb) (Fig. 2). Another feature of this clone was the copy number losses that appeared to the right of the rDNA locus of ChrR. WGS showed that during evolution in the drug, T1FM increased the copy number of the rDNA locus, albeit transiently, as the chromosomal amplifications disappeared in the highly resistant T1FH strain. Thus, our results support the observations that the rDNA locus is a fragile site that can cause copy number variation on chromosome ends (25, 26) and additionally might be relevant for a rapid adaptation to fluconazole.

### Gene expression deregulation induced by mistranslation.

**FIG 3:**
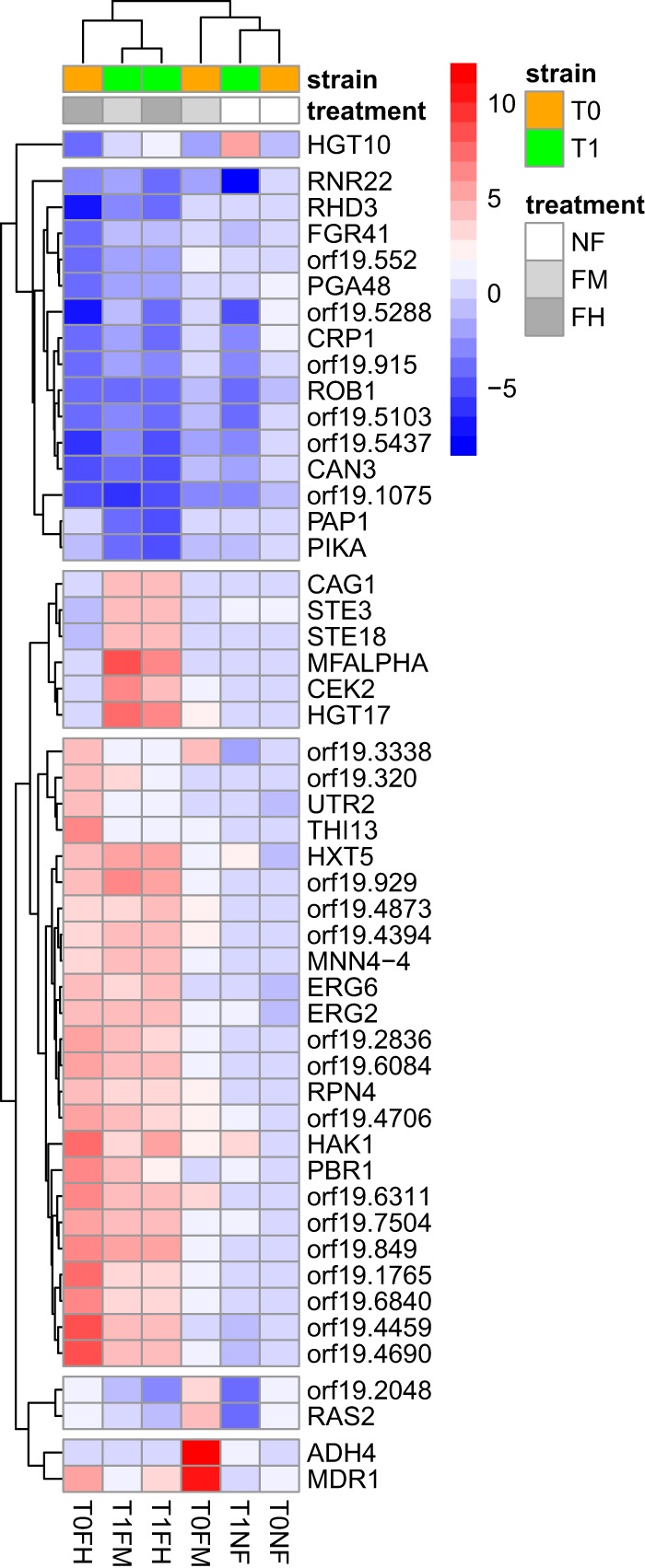
Gene expression heatmap. Depicted are DEGs with a 4-log-fold change for at least one comparison and a false discovery rate-adjusted *P* value of <1e−18.

Comparing the gene expression patterns of all of the strains and conditions used in this study revealed that during evolution in the drug, strain T0 showed a fluconazole-induced stress response, while strain T1 upregulated several mating-related genes, as well as genes encoding sugar transporters (Fig. 3). To distinguish the effects of adaptation to fluconazole from the effects of mistranslation, we compared the mRNA expression patterns of the hypermistranslating T1 strain that evolved in the presence (T1FH) and absence of the drug (T1NF) (Fig. S5). Gene set enrichment analysis (GSEA) (27) of T1FN detected deregulation of expression of translation genes, including those involved in tRNA and rRNA processing, tRNA methylation and modification, mRNA splicing, cytosolic and mitochondrial ribosomal subunit biosynthesis, and ribosomal biogenesis and assembly (Fig. S5). In T1FH, treatment with fluconazole resulted in downregulation of genes related to ribosome biogenesis, e.g., those required for maturation of large-subunit rRNA genes (Fig. S5). These GSEA results suggest that the hypermistranslating strain (T1) evolved in the absence of fluconazole had remodeled tRNA and rRNA processing and that addition of fluconazole repressed protein synthesis (Fig. S5). Mistranslation also upregulated genes involved in organic acid and amino acid metabolic processes and downregulated genes involved in glycoside and carbohydrate metabolic processes, cellular iron homeostasis, oxidation-reduction processes, and glycolysis (Fig. S6). The *FMP45* gene, which is involved in sensitivity to toxic ergosterol analogs and is induced during mating, was downregulated during evolution of the hypermistranslating T1 strain in the absence of fluconazole (T1NF) but upregulated in the presence of the drug (T1FH) (Fig. S6), suggesting that *FMP45* may also play a role in fluconazole resistance.

10.1128/mSphere.00167-17.5 FIG S5  Gene set enrichment networks. (A) Network displaying gene sets enriched in hypermistranslating strain T1FH relative to strain T0FH evolved in the presence of fluconazole. (B) Network displaying gene sets enriched in strain T1 evolved in the presence of a high fluconazole concentration (T1FH) relative to strain T1 evolved without the drug (T1NF). (C) Network displaying gene sets enriched in hypermistranslating strain T1 evolved without the drug (T1NF) versus nonevolved strain T1. Edge width represents the number of shared genes, node size represents the number of annotated genes, and node color represents the enrichment based on the number of upregulated (red) or downregulated (blue) genes in the set. The 10 genes ranked highest in each gene set are shown. Download FIG S5, PDF file, 0.9 MB. Copyright © 2017 Weil et al. 2017 Weil et al. This content is distributed under the terms of the Creative Commons Attribution 4.0 International license.

10.1128/mSphere.00167-17.6 FIG S6  Genes expressed differentially in T1FH and T1NF and functional analysis. (Top) Venn diagram in which each colored area represents a set of DEGs (differential expression threshold of ≥3.0, adjusted *P* value of <0.01). Only one gene, *FMP45*, is significantly upregulated in T1FH and downregulated in T1NF. The top two GO terms for each condition are shown. (Bottom) GO BP categories enriched below a *P* value of 0.01 in all of the expression sets shown in the Venn diagram. Download FIG S6, PDF file, 1 MB. Copyright © 2017 Weil et al. 2017 Weil et al. This content is distributed under the terms of the Creative Commons Attribution 4.0 International license.

### Mating and cell surface alterations.

**FIG 4:**
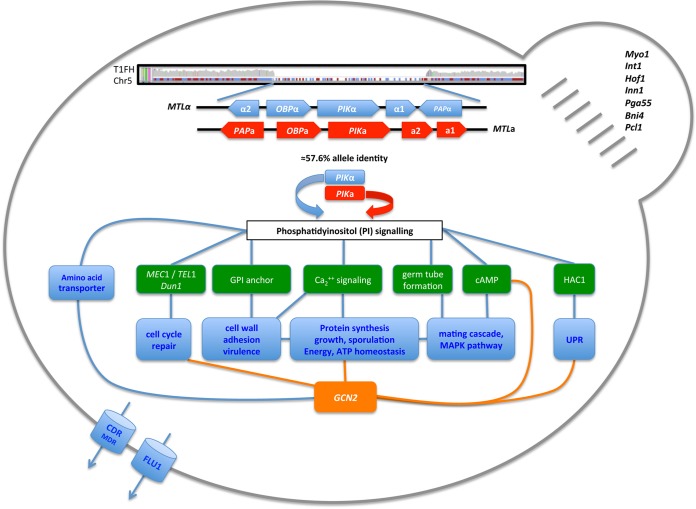
Highlighted features of quickly evolving and highly resistant strain T1. Mistranslation appears to affect the phosphatidylinositol signal transduction pathways that control several cell cycle events, cell membrane and cell wall remodeling, as well as protein synthesis and turnover, via *GCN2*, most of which are known to enhance drug resistance. The main features of highly drug-resistant strain T1FH are depicted, i.e., loss of *MTLa* and the nonsex gene *PIKa*; phosphatidylinositol signaling and downstream targets such as DNA repair, cell wall remodeling, and mating; *GCN2*-mediated modulation of protein synthesis; drug efflux pumps; and bud neck formation.

WGS and CGH also showed that T1 lost the *MTLa* locus (including the nonsex genes *PAP1*, *PIKa*, and *OBPa*) during evolution in fluconazole (Fig. 2 and 4; Fig. S7) and increased the copy number of several genes on Chr7 involved in mating, transport, and translation, including orf19.6583, orf19.7038, *FLU1*, *WHI3*, *TOM70*, *NBP2*, *YCF1*, and *GCN2* (Table 1).

10.1128/mSphere.00167-17.7 FIG S7  (A) Flow cytometry profiles of two T1FH clones. Red, diploid control; blue, sample. The T1FH profile indicates a mixed population of diploids, tetraploids, and some cells with greater ploidy. (B) Confirmation of loss of the *MTLa* locus in T1FH by targeted PCR. Colony PCR products of the *MTLa1* (red) and *MTLalpha1* (blue) genes visualized on an agarose gel. *MTLa1* is absent from all tested clones of the evolved and highly resistant strain T1FH. For the sequences of the *MTLa* primers used, see I. Miranda, R. Rocha, M. C. Santos, D. D. Mateus, G. R. Moura, L. Carreto, and M. A. Santos, PLoS One 2:e996, 2007. Download FIG S7, EPS file, 0.4 MB. Copyright © 2017 Weil et al. 2017 Weil et al. This content is distributed under the terms of the Creative Commons Attribution 4.0 International license.

**TABLE 1:**
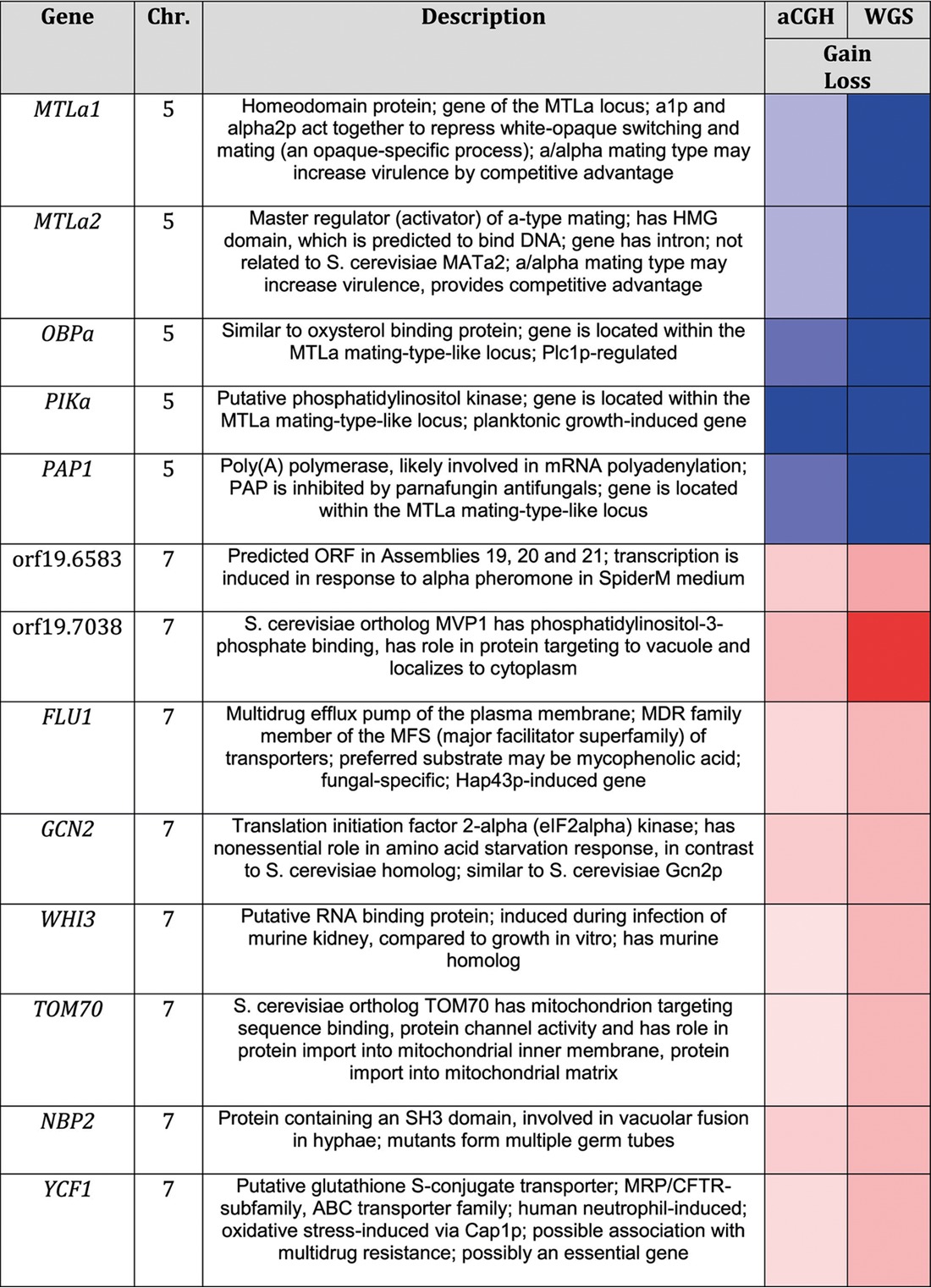
Copy number variation in T1FH^a^

a Genes that revealed high concordance in copy number changes in both SNP-CGH and WGS analysis are shown. Apart from the MTL genes that were also lost in T1FM, all of the gene alterations listed appeared exclusively in strain T1FH. Colors: red, copy number gain; blue, copy number loss. Increasing color intensity reflects an increase in gain or loss, respectively.

In addition to those genome alterations, in T1FH, we observed a 6.5-fold increase in the expression of *MFalpha*, the alpha factor mating pheromone gene, and a gene set enrichment of mating-specific terms like bud neck and ascospore formation, pheromone-dependent transcription involved in conjugation with cellular fusion, and terms related to virulence, like adhesion to a host and immune response (Fig. 3; Fig. S6). These gene expression alterations in T1FH are possibly related to loss of the *MTLa* locus and/or the LOH of Chr5 (28–30).

## DISCUSSION

Mistranslation is linked to elevated mutagenesis and the appearance of drug resistance in bacteria (17, 31), but its role in eukaryotes is still poorly understood. In this study, we used experimental evolution to examine the ability of a *C. albicans* strain that mistranslates at high levels to acquire resistance to fluconazole. By comparing changes in a hypermistranslating strain with those in a WT strain subjected to or excluded from fluconazole treatment, we could detect contributions of mistranslation to drug resistance at both the genome and transcription levels. During evolution in the absence of fluconazole, the resistance to fluconazole of both the WT T0 and hypermistranslating T1 strains was similar. In contrast, strain T1 became resistant to fluconazole at a much earlier time point during evolution in the drug. Our data suggest that different ways of upregulating drug efflux pumps, together with genomic changes in drug target genes, may speed up the acquisition of resistance in mistranslating strains. Increased drug efflux in the T0FM strain was likely mediated via the multidrug efflux pump encoded by *MDR1*, while T1FM overexpressed the ABC superfamily transporter genes *CDR1* and *CDR2*. Additionally, in T1FM, *ERG11*, the gene encoding the fluconazole target protein, became homozygous. An elevated copy number of the *FLU1* gene and the open reading frame for a putative multidrug resistance protein (orf19.3218) may also have contributed to fluconazole resistance in T1.

Recent literature suggests that a common mechanism for the acquisition of drug resistance involves LOH, which is often accompanied by aneuploidy (11). In T1FH, the two major LOH regions of ChrR and Chr5 include a large proportion of genes involved in both translation and drug resistance. This suggests that allele-specific gene functions may contribute to rapid adaptation to azoles and/or to stabilization of the mistranslation state and that CTG codon usage seems to have a balancing effect on such genomic events (Table S2). Further, the tRNA aminoacylation system might be a driving force for the LOH in hypermistranslators.

Interestingly, mistranslation led to both upregulation of amino acid metabolism and mutations in tRNA synthetases (T1NF), with the latter being increased under drug pressure (T1FH). For example, *PIKa* encodes a phosphatidylinositol kinase and is located within the *MTLa* locus that was lost on Chr5. While Δ*MTLa1* Δ*MTLa2* allows growth even in the highest levels of fluconazole (32), Δ*PIKa* may play a role in translation initiation. In *Saccharomyces cerevisiae* conditional mutants of the *PIKa* ortholog, *Pik1* controls translation initiation independently of *TOR* (33). *S. cerevisiae Pik1* mutation leads to a 4-fold increase in the phosphorylation of translation initiation factor 2 alpha (*elf2*α). Further, the general amino acid control (GCN) response induces the amino acid biosynthesis pathways upon the accumulation of uncharged tRNAs (34, 35). The increase in the copy number of *elf2*α kinase *GCN2* observed in the T1FH strain suggests that high levels of *elf2*α phosphorylation could be a specific translational modification required for adaptation to fluconazole.

Another feature that appeared in T1 during drug resistance evolution was the accumulation of a large number of SNPs and codon changes in the *PGA*, *IFF*, and *ALS* genes, which encode glycosyl-phosphatidylinositol (GPI)-anchored proteins. Additionally, the *AGM1* gene, which is involved in the chitin biosynthetic pathway (synthesis of UDP-*N*-acetylglucosamine), was specifically upregulated in evolved T1 strains (Fig. S3). *N*-Acetylglucosamine is a moiety of the synthesis of GPI anchors and a building block of chitin (36). Sequences encoding GPI-anchored adhesins are rich in CUG codons, and a recent study demonstrated that CUG mistranslation enhances the ability of *C. albicans* to adhere to different surfaces, especially under stress conditions (37). Remodeling of the cell wall and modification of adhesion properties are important features of drug resistance and virulence (38, 39). Consistent with this, T1FH flocculated and showed strong adhesion to surfaces in liquid medium (data not shown). Hence, by expanding the variability of cell wall proteins, CUG codon ambiguity could allow *C. albicans* to accelerate the acquisition of antifungal resistance.

Several serine/threonine kinases and serine/threonine phosphatases lost heterozygosity and/or changed gene expression levels in T1FH. Here, overexpression of the *SHA3* gene, encoding a Ser/Thr kinase, which regulates glucose transport, is likely responsible for the upregulation of sugar import genes in the resistant T1FH strain. Members of the transporter-encoding *HGT* and *HXT* gene families were also overexpressed. As the genes regulating the first steps of glycolysis (*PGI1*, *PFK1*, and *PFK2*) were downregulated and *INO1*, which encodes the key enzyme of *myo*-inositol biosynthesis, was upregulated, we assume that the imported glucose is channeled into *myo*-inositol biosynthesis in T1FH, which is essential for growth and virulence in *C. albicans* (40). Additionally, the *Mec1* gene, which is located on the right arm of Chr5, was one of the few genes that were highly enriched in LOH events (*Mec1*, 60 [median, 5] LOH events) exclusively in T1 strains exposed to fluconazole. *Mec1* is a serine threonine- and leucine-rich putative phosphatidylinositol kinase that, upon DNA damage, phosphorylates a large set of proteins, and together with its yeast ortholog, *Mec1* was repeatedly linked to genome instability, telomere maintenance, and drug response (3, 41, 42). Flow cytometry (Fig. S7) suggested that T1FH cells have multiple ploidy states, including diploids, tetraploids, and a smaller population of cells with >4c ploidy. Alterations in ploidy were recently shown to occur soon after *C. albicans* cells are exposed to fluconazole, and it is thought that they provide the first step in the formation of aneuploid cells with a wide range of phenotypic variability (43).

Taken together, our data suggest that mistranslation mediated more rapid evolution of fluconazole resistance via a range of mechanisms, including the classical effects on efflux and ergosterol biosynthesis, among others. Dissection of the genes necessary and sufficient for this evolution will require additional studies. In particular, parallel evolution of multiple clones of both hypermistranslating and WT strains is necessary to clarify the adaptive and mechanistic relevance of the multiple and important mutations identified in this study. Our data leave no doubt that mistranslation accelerates the evolution of resistance to fluconazole in *C. albicans*; whether mistranslation is a major driver of mutagenesis in drug stress will be fascinating to study in future work.

## MATERIALS AND METHODS

### Strains and growth conditions.

The *C. albicans* strains used in this study were engineered to exhibit different levels of leucine misincorporation at serine CUG codons. The T0 control strain incorporates 3.2% Leu and 96.8% Ser at CUG codons (normal level of mistranslation in *C. albicans*); T1 incorporates 22.5% Leu and 77.5% Ser at CUG codons (16). All strains were constructed with the SN148 parental strain (*arg4*Δ/*arg4*Δ *leu2*Δ/*leu2*Δ *his1*Δ/*his1*Δ *ura3*Δ::*imm43*/*ura3*Δ::*imm43 iro1*Δ::*imm43*/*iro1*Δ::*imm43*) (44) by homologous recombination as described in reference 16.

Incorporation of Leu and Ser at CUG codons was determined with a previously developed gain-of-function reporter (16). Fluorescence was quantified with a Zeiss MC80 Axioplan 2 light microscope equipped for epifluorescence microscopy with the HE38 filter set (Carl Zeiss AG). Images were taken with an AxioCam HRc camera and analyzed with ImageJ software (https://imagej.nih.gov/ij/) as previously described (16).

Strains were grown in synthetic defined (SD) medium without uracil containing 0.67% yeast nitrogen base, 2% glucose, and 0.2% dropout mix (with 2% agar for solid medium only) at 30°C.

### Experimental evolution.

Resistance to fluconazole was monitored by the E-test method (AB Biodisk, BioMérieux). Cells were grown to mid-log phase, washed in 1× phosphate-buffered saline, and diluted to an optical density at 600 nm of 0.015. A 150-μl volume of cells was plated on SD minus uracil agar plates (pH 7) with glass beads and allowed to dry for 15 to 30 min before a fluconazole E-test strip (0.016 to 256 μg/ml; AB Biodisk) was applied. Plates were incubated at 30°C for 48 h, and the MIC was determined as the concentration at which the first growth inhibition ellipse occurred. To perform the evolution and E-test experiments in parallel, cells were incubated in liquid (SD minus uracil) medium adjusted to 1× and 2× the last measured fluconazole (Sigma-Aldrich) MIC. Depending on growth in the respective concentrations of fluconazole, passages were pursued until strains were able to grow in liquid medium containing a fluconazole concentration of 256 μg/ml (Fig. S1). Additionally, control clones were evolved for an equal number of passages without fluconazole to study the effects of mistranslation and the antifungal (T0NF and T1NF).

### Nucleic acid extraction.

RNA and DNA were extracted from strains at the beginning (T0 and T1), middle (T0FM and T1FM), and end of evolution with fluconazole (T0FH and T1FH) and from the evolved control strains (T0NF and T1NF) (Fig. S1). Additionally, to test for clonal diversity, resistant strains were grown in the highest concentration plated on SD minus uracil agar plates and three single colonies (T1FHa to T1FHc) were picked for DNA extraction.

RNA was extracted from mid-exponential-phase cells by the hot phenol method. DNase I (Invitrogen)-treated RNA was resuspended in RNase-free water, and RNA quantity and integrity were assessed by UV/Vis spectrometry (NanoDrop; Thermo Scientific) and with the Agilent 2100 Bioanalyzer, respectively. Only RNAs with an integrity number of >9 were selected for further analysis.

DNA was extracted with the Genomic-tip 100/G kit (Qiagen) in accordance with the manufacturer’s instructions. DNA was resuspended in EB buffer, and DNA quantity and integrity were assessed with the Quant-iT PicoGreen double-stranded DNA quantitation assay and by agarose gel electrophoresis.

### Transcriptome analysis.

Custom gene expression SurePrint G3 microarrays (Agilent-065138) were hybridized with CY3-labeled cRNA of growth duplicates in accordance with the manufacturer’s instructions (Agilent Low Input Quick Amp Labeling kit). Arrays were scanned on an Agilent G2505C microarray scanner, and microarray scan data were extracted with Agilent feature extraction software.

Raw gene expression microarray data were normalized with limmaGUI software (R/Bioconductor, Boston, MA) (45). Differentially expressed genes (DEGs) were extracted with limma (45) by using a differential threshold of 1.5 and a false discovery rate-corrected *P* value of 0.05. Functional enrichment was performed with GSEA (27) based on GO terms. Strains evolved in the presence of fluconazole (TxFM, TxFH) were initially compared to the respective untreated and nonevolved strain (Tx). Additionally, TxFH strains were compared among themselves and against the respective control strain (TxNF). For each comparison, GO sets with >15 and <500 annotated genes were selected as enriched if the corrected *P* value was <0.01 (1,000 gene set permutations). Enriched sets were related upon shared genes with the Cytoscape plugin EnrichmentMap (46). Figure 3 was generated with the R package pheatmap (47). Voronto software (48) was used for visualization of gene expression on the basis of GO biological process (BP) categories (Fig. S3).

### Whole-genome resequencing.

One paired-end library was prepared for each sample in accordance with Illumina DNA sample preparation protocols. Libraries were sequenced with the Illumina genome analyzer IIx.

Raw sequence data (101- and 146-bp paired-end reads) from eight samples (T0, T1, T0FM, T1FM, T0FH, T1FH, T0NF, and T1NF) were initially trimmed by removing consecutive bases on both the 5′ and 3′ flanks with base qualities of <20. Trimmed reads that did not pass the filtering criteria for ambiguity (N content of <5%), complexity (score of ≥10), length (50 bases or longer), and average base quality (≥20) were removed with PRINSEQ (49).

Remaining reads were mapped to the reference genome of *C. albicans* obtained from the *Candida* genome database (http://www.candidagenome.org/; assembly 21) with BWA version 0.6.2 (50). Processing and filtering of mapped reads were done with SAMtools version 0.1.17 (51). After removal of duplicates, read pairs where both reads mapped on different chromosomes were removed. Additionally, read pairs where one or both reads were not mapped or had low mapping quality (<37) were removed.

The control strain used in this experiment, T0, is genetically slightly different from SC5314, which was used to construct the reference genome. SAMtools was used to produce read pileups, detect single nucleotide variants, and call genotypes. Indels were not called. Bases with a read depth of <5, a low quality of REF/ALT polymorphism (<20), low genotype quality (<20), and low consensus quality (<|20|) were called as unknown genotype. Additionally, unknown genotypes were called if a base had more than three allele types and the read depths of all of the allele types were >5.

For the eight samples, we analyzed genomic features (coding, noncoding, or repeat region, etc.) and LOH of bases with different genotype compared with the control strain. Structural variations in coding regions were also analyzed for changes in codon usage.

Copy number analysis was performed with CNAnorm (52). Aligned reads of each sample were split into 2-kb windows, and reads were counted. CNAnorm was used to estimate copy numbers with normalization of paired control and test samples.

### SNP-CGH.

SNP-CGH array analysis was performed with custom Agilent arrays (eArray Design ID 038464 [53]). Three T1FH clones (T1FHa to T1FHc) were compared with control strain T0. Genomic DNA was digested with AluI and RsaI, amplified, labeled, and hybridized in accordance with the manufacturer’s instructions with the Agilent Genomic DNA enzymatic labeling kit (Agilent). Arrays were scanned on an Agilent G2505C microarray scanner, and microarray scan data were extracted with Agilent feature extraction software. All of the samples analyzed in this study passed the quality control performed with the R/Bioconductor packages GLAD (positional biases) and arrayQualityMetrics (statistical distributions) without any relevant issue (54, 55). Genomic events for SNP-CGH data were predicted by computing hidden Markov model states via the R/Bioconductor SNP-CGH package (56) by using default parameters. Genome ratios were plotted with the R/Bioconductor SNP-CGH package and mapped against *C. albicans* chromosome annotations (http://www.candidagenome.org/; assembly 21). Allele fractions were calculated with a custom R script from the SNP probes in the SNP-CGH data.

Additionally, the data were visualized with Y_MAP_ (57). Therefore, the array data files produced during extraction were uploaded into Y_MAP_ for processing by using the SNP-CGH array processing option with the GC bias correction option selected to correct for artifacts in the array data caused by variations in percent GC across the genome. After Y_MAP_ processed the data, it produced cartoon figures displaying copy number and allelic ratio changes across the genome for each clone. See the legend to Fig. 2 for details of how the data are presented and for interpretation of the data for each of the three clones (T1FHa to T1FHc).

### Accession number(s).

The raw microarray data obtained in this study have been submitted to the Gene Expression Omnibus and assigned GenBank accession numbers GSE60121 and GSE60122. The array CGH data are listed in the Gene Expression Omnibus under GenBank accession number GSE60120. Sequencing data were archived in ArrayExpress under accession number E-SYBR-13.
