# Supplementary material for: Adaptive Mistranslation Accelerates the Evolution of Fluconazole Resistance and Induces Major Genomic and Gene Expression Alterations in Candida albicans
Source: mSphere. 2017 Aug 9;2(4):e00167-17. doi: 10.1128/mSphere.00167-17 (PMC5549176; doi:10.1128/mSphere.00167-17)
Supplement: TABLE S1 [file sph004172333st8.docx]

| **Strain** | **N.ofAA** | **T0.Codon** | **T0.AA** | **S.Codon** | **S.AA** |
| --- | --- | --- | --- | --- | --- |
| *TAC1* | | | | | |
| T1FH | 736 | GCA | A | GTA | V |
| *MRR1* | | | | | |
| T0FM | 877 | GTT | V | TTT | F |
| T0FM | 943 | TTG | L | TTC | F |
| *ERG7* | | | | | |
| T0FM | 35 | TCT | S | TGT | C |
| T0FH | 35 | TCT | S | TGT | C |
| T1FM | 35 | TCT | S | TGT | C |
| T1NF | 35 | TCT | S | TGT | C |
