# Supplementary material for: Adaptive Mistranslation Accelerates the Evolution of Fluconazole Resistance and Induces Major Genomic and Gene Expression Alterations in Candida albicans
Source: mSphere. 2017 Aug 9;2(4):e00167-17. doi: 10.1128/mSphere.00167-17 (PMC5549176; doi:10.1128/mSphere.00167-17)
Supplement: TABLE S2 [file sph004172333st9.pdf]

Table S2A

T1 LOH							
	AGC	AGU	UCA	UCC	UCG	UCU	CUG
**Average altered**	0.27	1.55	0.46	0.55	0.34	1.61	0.23
**Average other genes**	0.38	0.37	**2.08**	**0.74**	**0.52**	1.59	0.32
**SD altered**	0.30	0.63	0.73	0.31	0.25	0.68	0.24
**SD other genes**	0.41	0.71	0.85	0.59	0.50	0.85	0.39
**N° altered**	62	62	62	62	62	62	62
**N° other genes**	6007	6007	6007	6007	6007	6007	6007
**Pvalue**	0.004	0.028	0.000	0.000	0.000	0.817	0.007
**Bonferoni (*7)**	0.030	0.198	0.001	0.000	0.000	5.722	0.050
**T1FH LOH**							
	AGC	AGU	UCA	UCC	UCG	UCU	CUG
**Average altered**	0.36	1.42	2.16	0.69	0.45	1.59	0.32
**Average other genes**	0.38	1.37	2.08	0.74	**0.53**	1.59	0.32
**SD altered**	0.38	0.64	0.80	0.50	0.36	0.78	0.35
**SD other genes**	0.42	0.72	0.86	0.60	0.51	0.86	0.40
**N° altered**	650	650	650	650	650	650	650
**N° other genes**	5419	5419	5419	5419	5419	5419	5419
**Pvalue**	0.255	0.041	0.020	0.021	0.000	0.772	0.708
**Bonferoni (*7)**	1.000	0.284	0.143	0.147	0.000	1.000	1.000
**T1FH CNV**							
	AGC	AGU	UCA	UCC	UCG	UCU	CUG
**Average altered**	0.26	1.60	**2.40**	0.53	0.35	1.49	0.37
**Average other genes**	0.38	1.37	2.08	**0.74**	**0.52**	1.59	0.32
**SD altered**	0.40	0.73	0.98	0.52	0.47	0.82	0.37
**SD other genes**	0.41	0.71	0.85	0.59	0.50	0.85	0.39
**N° altered**	110	110	110	110	110	110	110
**N° other genes**	5959	5959	5959	5959	5959	5959	5959
**Pvalue**	0.003	0.001	0.001	0.000	0.000	0.197	0.155
**Bonferoni (*7)**	0.023	0.010	0.007	0.000	0.002	1.000	1.000

Table S2B

T1FH LOH					
**Orf**	**Chr**	**Name**	**Allele a**	**Allele b**	**Diff a vs b**
orf19.2604	chrR		**3**	0	3
orf19.3214	chr5		**2**	0	2
orf19.3724	chrR		**1**	0	1
orf19.2391	chrR		**4**	3	1
orf19.3737	chrR		**3**	2	1
orf19.1116	chr5		**6**	5	1
orf19.2400	chrR		**7**	6	1
orf19.2616	chrR	UGT51C1	**4**	3	1
orf19.2401	chrR		**7**	6	1
orf19.3178	chr5		**4**	3	1
orf19.4251	chr5	ZCF22	**6**	5	1
orf19.3188	chr5	TAC1	**5**	4	1
orf19.4369	chrR		**12**	11	1
orf19.4131	chr5		**6**	5	1
orf19.1283	chr5	MEC1	**13**	12	1
orf19.935	chr5	AGA1	3	**4**	-1
orf19.3919	chr5		4	**5**	-1
orf19.1121	chr5		0	**1**	-1
orf19.3942	chr5		1	**2**	-1
orf19.4279	chr5	MNN1	2	**3**	-1
orf19.6297	chr5		3	**4**	-1
orf19.4401	chrR	YVH1	3	**4**	-1
orf19.1971	chr5		1	**2**	-1
orf19.4221	chr5	ORC4	2	**3**	-1
orf19.2832	chrR	INN1	5	**6**	-1
orf19.2839	chrR	CIRT4B	3	**4**	-1
orf19.3204	chr5		4	**5**	-1
orf19.3906	chr5		1	**2**	-1
orf19.2540	chrR	SAS3	6	**7**	-1
orf19.932	chr5		3	**4**	-1
orf19.2521	chrR		8	**9**	-1
orf19.4346	chr5		12	**13**	-1
orf19.3190	chr5	HAL9	6	**7**	-1
orf19.1958	chr5		0	**2**	-2
orf19.3726	chrR		5	**7**	-2
orf19.4257	chr5	INT1	5	**7**	-2

T1FH CNV					
**Orf**	**Chr**	**Name**	**Allele a**	**Allele b**	**Diff a vs b**
orf19.2371	chr7	GAG1	7	6	1
orf19.2372	chr7	POL99	7	6	1
orf19.3699	chr7	TEP1	1	0	1
orf19.6458	chr7		2	1	1
orf19.6469	chr7		13	12	1
orf19.5775	chr6		4	5	-1
