# Supplementary material for: Adaptive Mistranslation Accelerates the Evolution of Fluconazole Resistance and Induces Major Genomic and Gene Expression Alterations in Candida albicans
Source: mSphere. 2017 Aug 9;2(4):e00167-17. doi: 10.1128/mSphere.00167-17 (PMC5549176; doi:10.1128/mSphere.00167-17)
Supplement: TABLE S3 [file sph004172333st10.docx]

| **GOID** | **GO BP** | **Corrected P-value** |
| --- | --- | --- |
| 35672 | oligopeptide transmembrane transport | 0.00021 |
| 6857 | oligopeptide transport | 0.00113 |
| 6418 | tRNA aminoacylation for protein translation | 0.00121 |
| 90304 | nucleic acid metabolic process | 0.00135 |
| 9987 | cellular process | 0.00154 |
| 43038 | amino acid activation | 0.00163 |
| 43039 | tRNA aminoacylation | 0.00163 |
| 44699 | single-organism process | 0.00180 |
| 19222 | regulation of metabolic process | 0.00260 |
| 44763 | single-organism cellular process | 0.00359 |
| 6348 | chromatin silencing at telomere | 0.00392 |
| 15833 | peptide transport | 0.00411 |
| 34641 | cellular nitrogen compound metabolic process | 0.00453 |
| 1901360 | organic cyclic compound metabolic process | 0.00474 |
| 19219 | regulation of nucleobase-containing compound metabolic process | 0.00519 |
| 31323 | regulation of cellular metabolic process | 0.00525 |
