# Supplementary material for: Adaptive Mistranslation Accelerates the Evolution of Fluconazole Resistance and Induces Major Genomic and Gene Expression Alterations in Candida albicans
Source: mSphere. 2017 Aug 9;2(4):e00167-17. doi: 10.1128/mSphere.00167-17 (PMC5549176; doi:10.1128/mSphere.00167-17)
Supplement: FIG S5 [file sph004172333sf5.pdf]

Fig. S5A

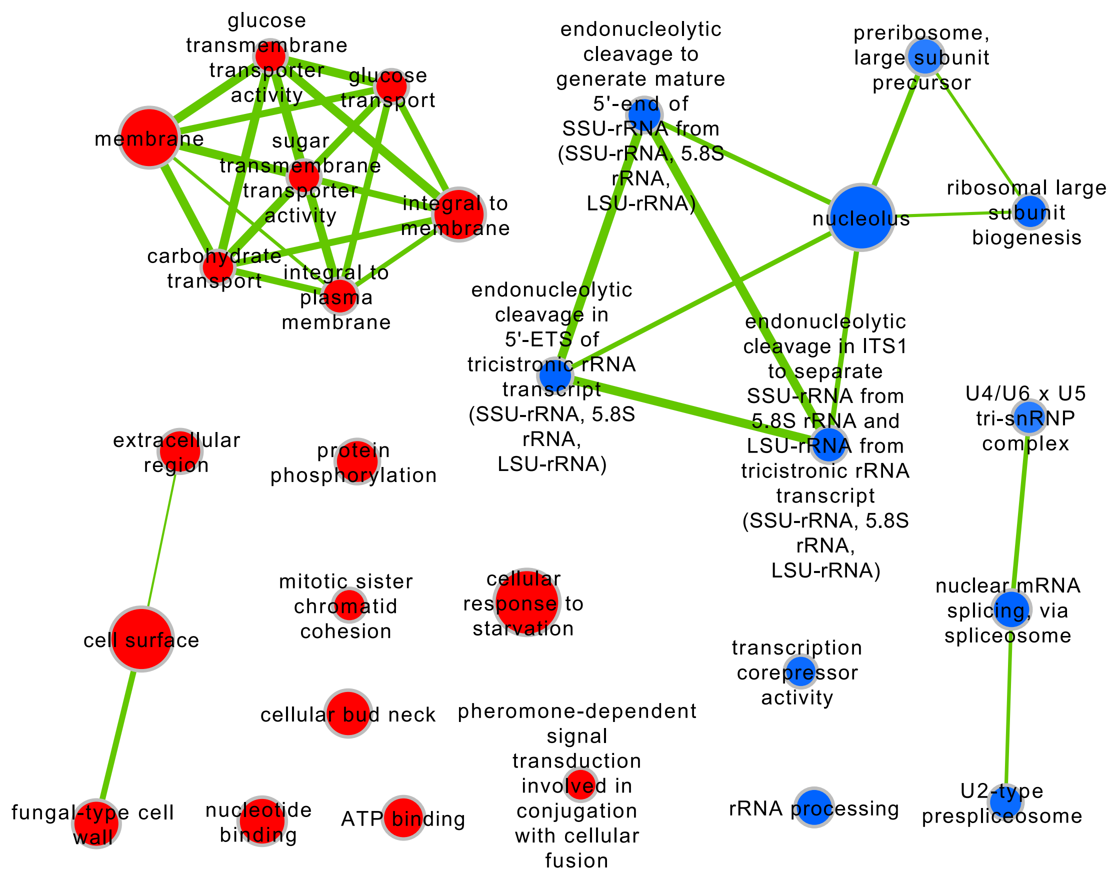

Fig. S5B

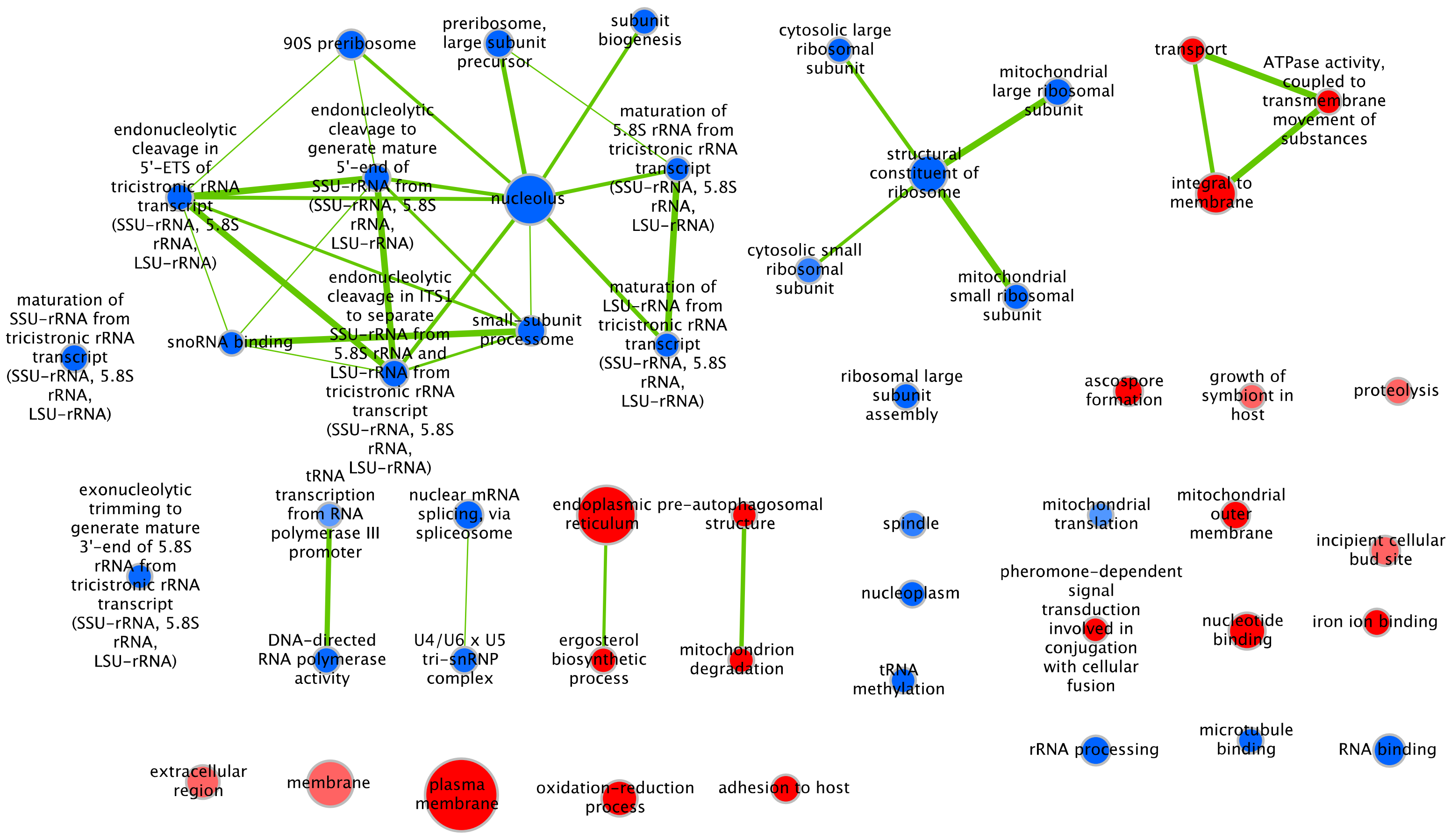

Fig. S5C

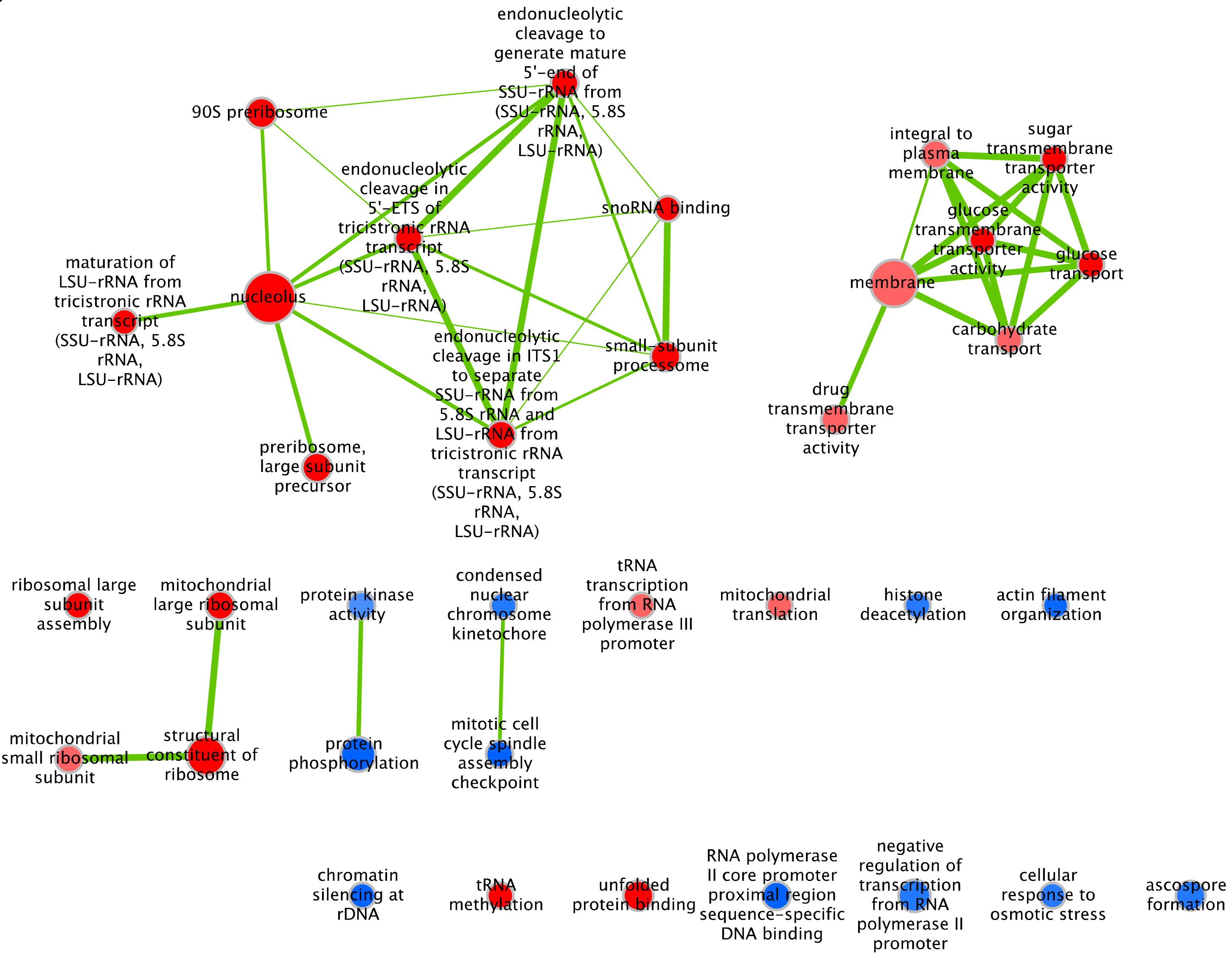

Top10 ranked gene list for Networks:

T1FH vs T0FH<br>Fig.S5A	T1FH vs T1NF<br>Fig.S5B	T1NF vs T1<br>Fig.S5C
MFALPHA	FRP1	orf19.4342
orf19.4653	MFALPHA	orf19.6487
CEK2	orf19.1691	orf19.341
STE3	ADH3	LDG3
HGT17	orf19.265	orf19.3908
orf19.552	orf19.849	MET3
HGT10	HGT17	HGT10
orf19.7056	CEK2	orf19.6484
orf19.1736	ATO9	HGT12
PSO2	orf19.929	PGA31
THI4	OBPA	orf19.725
PBR1	AAH1	MYO1
orf19.7283	RHD2	orf19.2629
GTT12	HNM3	orf19.6982
OBPA	PIKA	RSN1
HAL9	MTLA1	orf19.188
orf19.7330	PGA31	orf19.1691
PAP1	PAP1	AQY1
PIKA	HAL9	PLB1
MTLA1	orf19.6487	RNR22
