# Supplementary material for: Adaptive Mistranslation Accelerates the Evolution of Fluconazole Resistance and Induces Major Genomic and Gene Expression Alterations in Candida albicans
Source: mSphere. 2017 Aug 9;2(4):e00167-17. doi: 10.1128/mSphere.00167-17 (PMC5549176; doi:10.1128/mSphere.00167-17)
Supplement: FIG S6 [file sph004172333sf6.pdf]

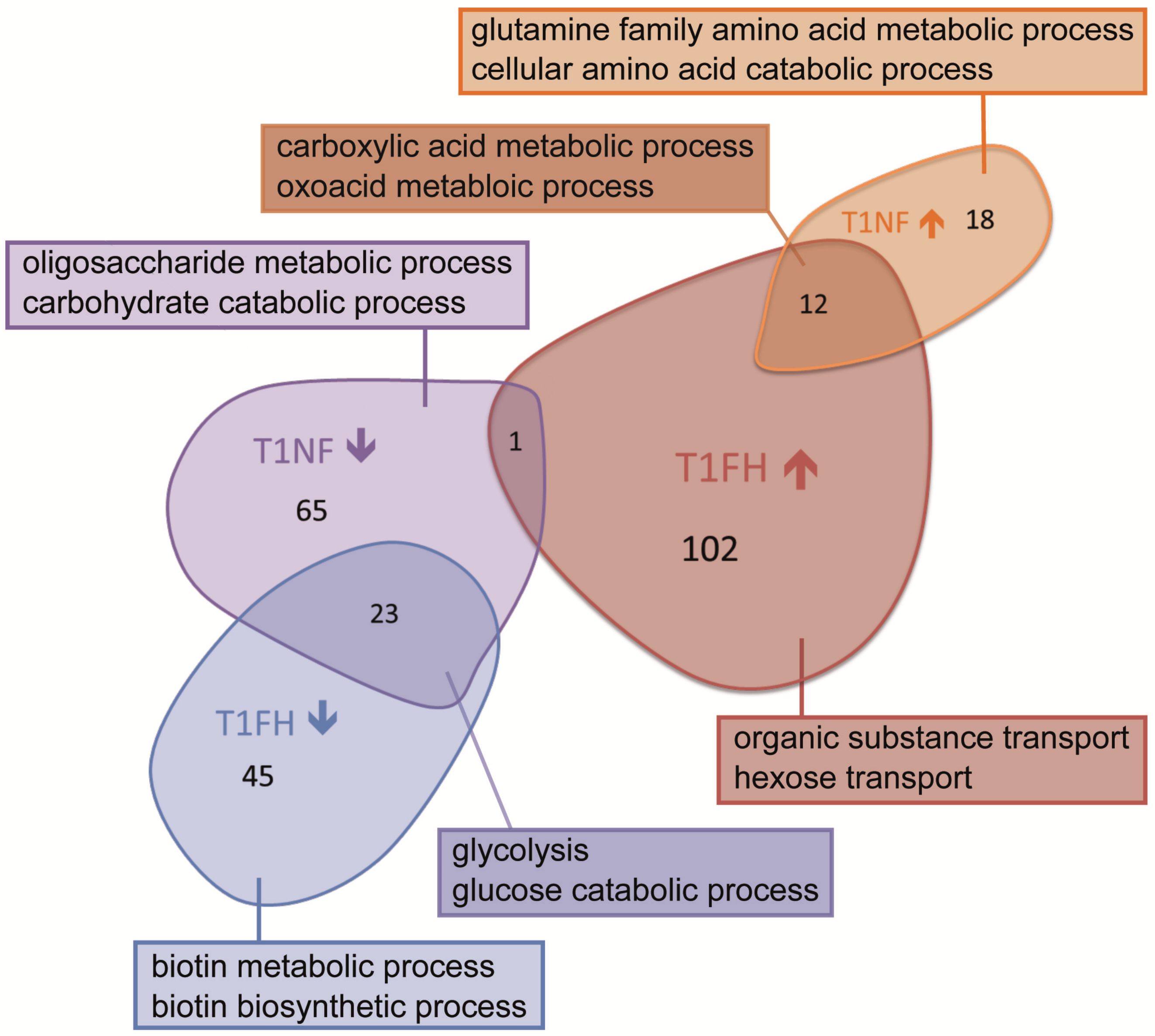

GO.ID	Term	p-value	dgenes
	**down-regulated in T1NF and T1FH**		
GO:0006096	glycolysis	0.0038	GPM2, PFK1
GO:0006007	glucose catabolic process	0.0089	GPM2, PFK1
	**down-regulated in T1NF**		
GO:0009311	oligosaccharide metabolic process	0.00014	TPS1, AMS1, HSP21, orf19.4488
GO:0016052	carbohydrate catabolic process	0.00036	PFK2, GLK4, GPH1, AMS1, orf19.4488
GO:0005984	disaccharide metabolic process	0.00042	TPS1, HSP21, orf19.4488
GO:0016137	glycoside metabolic process	0.00042	TPS1, HSP21, orf19.4488
GO:0005975	carbohydrate metabolic process	0.00324	DAK2, PFK2, TPS1, GLK4, GPH1, AMS1, HSP21, orf19.4488
GO:0005991	trehalose metabolic process	0.00366	TPS1, HSP21
GO:0009313	oligosaccharide catabolic process	0.00366	AMS1, orf19.4488
GO:0055114	oxidation-reduction process	0.00402	OSM1, DES1, HEM13, SUR2, XYL2, ADH5, DOT5, GPH1, FRP1, IFD6
GO:0006879	cellular iron ion homeostasis	0.00450	PGA10, RBT5, FRP1
GO:0055072	iron ion homeostasis	0.00482	PGA10, RBT5, FRP1
GO:0044262	cellular carbohydrate metabolic process	0.00698	DAK2, TPS1, GPH1, HSP21, orf19.4488
	**down-regulated in T1FH**		
GO:0006768	biotin metabolic process	7,00E-06	orf19.2591, HNM3, orf19.2590
GO:0009102	biotin biosynthetic process	7,00E-06	orf19.2591, HNM3, orf19.2590
GO:0043604	amide biosynthetic process	7,00E-06	orf19.2591, HNM3, orf19.2590
GO:0043603	cellular amide metabolic process	0.00012	orf19.2591, HNM3, orf19.2590
GO:0051180	vitamin transport	0.00106	orf19.2397, HNM3
GO:0009110	vitamin biosynthetic process	0.00234	orf19.2591, HNM3, orf19.2590
GO:0042364	water-soluble vitamin biosynthetic process	0.00234	orf19.2591, HNM3, orf19.2590
GO:0006766	vitamin metabolic process	0.00272	orf19.2591, HNM3, orf19.2590
GO:0006767	water-soluble vitamin metabolic process	0.00272	orf19.2591, HNM3, orf19.2590
GO:0018130	heterocycle biosynthetic process	0.00445	CRP1, AAH1, orf19.2591, HNM3, orf19.2590
GO:0044272	sulfur compound biosynthetic process	0.00463	orf19.2591, HNM3, orf19.2590
GO:0044271	cellular nitrogen compound biosynthetic process	0.00681	CRP1, AAH1, orf19.2591, HNM3, orf19.2590
	**up-regulated in T1NF and T1FH**		
GO:0019752	carboxylic acid metabolic process	2.6e-05	IDP2, CIT1, MET3, HIS3, orf19.5565, MET14
GO:0043436	oxoacid metabolic process	2.6e-05	IDP2, CIT1, MET3, HIS3, orf19.5565, MET14
GO:0006082	organic acid metabolic process	2.6e-05	IDP2, CIT1, MET3, HIS3, orf19.5565, MET14
GO:0042180	cellular ketone metabolic process	3.9e-05	IDP2, CIT1, MET3, HIS3, orf19.5565, MET14
GO:0000103	sulfate assimilation	0.00010	MET3, MET14
GO:0055114	oxidation-reduction process	0.00012	IDP2, orf19.541, CIT1, MET3, orf19.5565, MET14
GO:0009086	methionine biosynthetic process	0.00070	MET3, MET14
GO:0006555	methionine metabolic process	0.00093	MET3, MET14
GO:0006520	cellular amino acid metabolic process	0.00098	MET3, HIS3, orf19.5565, MET14
GO:0000097	sulfur amino acid biosynthetic process	0.00129	MET3, MET14
GO:0044281	small molecule metabolic process	0.00129	IDP2, CIT1, MET3, HIS3, orf19.5565, MET14
GO:0009067	aspartate family amino acid biosynthetic process	0.00192	MET3, MET14
GO:0008652	cellular amino acid biosynthetic process	0.00201	MET3, HIS3, MET14
GO:0000096	sulfur amino acid metabolic process	0.00228	MET3, MET14
GO:0009066	aspartate family amino acid metabolic process	0.00282	MET3, MET14
GO:0044272	sulfur compound biosynthetic process	0.00387	MET3, MET14
GO:0016053	organic acid biosynthetic process	0.00474	MET3, HIS3, MET14
GO:0046394	carboxylic acid biosynthetic process	0.00474	MET3, HIS3, MET14
GO:0006790	sulfur compound metabolic process	0.00843	MET3, MET14
	**up-regulated in T1NF**		
GO:0009064	glutamine family amino acid metabolic process	0.00016	PUT1, GUA1, CAR1
GO:0009063	cellular amino acid catabolic process	0.00029	PUT1, GCV2, CAR1
GO:0009065	glutamine family amino acid catabolic process	0.00067	PUT1, CAR1
GO:0016054	organic acid catabolic process	0.00072	PUT1, GCV2, CAR1
GO:0046395	carboxylic acid catabolic process	0.00072	PUT1, GCV2, CAR1
GO:0044282	small molecule catabolic process	0.00129	PUT1, GCV2, CAR1
GO:0006520	cellular amino acid metabolic process	0.00499	PUT1, GUA1, GCV2, CAR1
	**up-regulated in T1FH**		
GO:0071702	organic substance transport	1.1e-05	CDR1, CDR2, HGT17, AAP1, HXT5, SFC1, orf19.7056, MDR1, HGT2, HGT1, NAG4, HGT19, MEP2
GO:0008645	hexose transport	1.2e-05	HGT17, HXT5, HGT2, HGT1, NAG4, HGT19
GO:0015749	monosaccharide transport	1.2e-05	HGT17, HXT5, HGT2, HGT1, NAG4, HGT19
GO:0008643	carbohydrate transport	3.4e-05	HGT17, HXT5, HGT2, HGT1, NAG4, HGT19
GO:0046618	drug export	4.8e-05	CDR1, CDR2, MDR1
GO:0006577	amino-acid betaine metabolic process	0.00017	CTN3, orf19.7131, CTN1
GO:0009437	carnitine metabolic process	0.00017	CTN3, orf19.7131, CTN1
GO:0015758	glucose transport	0.00080	HGT17, HGT2, HGT1, HGT19
GO:0055085	transmembrane transport	0.00186	CDR1, HGT17, AAP1, GEF2, HXT5, SFC1, orf19.7056, MDR1, FRP1, HGT2, HGT1, NAG4, HGT19, MEP2
GO:0006066	alcohol metabolic process	0.00188	CTN3, ERG6, CTN1, ERG27, ERG2, INO1, ATF1
GO:0015669	gas transport	0.00284	orf19.4459, YHB5
GO:0015671	oxygen transport	0.00284	orf19.4459, YHB5
GO:0070887	cellular response to chemical stimulus	0.00499	CDR1, SVF1, STE18, DAG7, CDR2, MFALPHA, CAG1, ERG6, RPN4, ERG27, STE3, ERG2, CIP1, MDR1, HGT1, NAG4, orf19.7029, MEP2
GO:0015893	drug transport	0.00563	CDR1, CDR2, MDR1, NAG4
GO:0000750	pheromone-dependent signal transduction involved in conjugation with cellular fusion	0.00603	STE18, CAG1, STE3
GO:0015850	organic alcohol transport	0.00769	CDR1, MDR1
GO:0050778	positive regulation of immune response	0.00769	HGT1, SAP4
GO:0032005	signal transduction involved in conjugation with cellular fusion	0.00773	STE18, CAG1, STE3
GO:0002684	positive regulation of immune system process	0.00978	HGT1, SAP4
GO:0006855	drug transmembrane transport	0.00978	CDR1, MDR1
GO:0006955	immune response	0.00978	HGT1, SAP4
GO:0050776	regulation of immune response	0.00978	HGT1, SAP4
